# Progress Toward Poliomyelitis Eradication — Nigeria, January 2012–September 2013

**Published:** 2013-12-13

**Authors:** Andrew Etsano, Faisal Shuaib, Pascal Mkanda, Richard Banda, Charles Korir, Melissa Corkum, Serigne Ndiaye, Samrawit Ashenafi, Frank Mahoney, John F. Vertefeuille, Cara C. Burns

**Affiliations:** National Primary Health Care Development Agency; Federal Ministry of Health, Federal Republic of Nigeria; World Health Organization, Nigeria Office; United Nations Children’s Fund, Nigeria Office; Global Immunization Div, Center for Global Health; Div of Viral Diseases, National Center for Immunization and Respiratory Disease, CDC

Transmission of wild poliovirus (WPV) has never been interrupted in Afghanistan, Pakistan, and Nigeria, and since 2003, Nigeria has been a reservoir for WPV reintroduction to 25 polio-free countries (1–3). In 2012, the Nigerian government activated an emergency operations center and implemented a national emergency action plan to eradicate polio (2,4). The 2013 revision of this plan prioritized 1) improving quality of supplemental immunization activities (SIAs), 2) implementing strategies to reach underserved populations, 3) adopting special approaches in security-compromised areas, 4) improving outbreak response, 5) enhancing routine immunization and activities implemented between SIAs, and 6) strengthening surveillance. This report summarizes polio eradication activities in Nigeria during January 2012–September 2013 and updates previous reports (2,5–7). During January–September 2013, 49 polio cases were reported from 26 local government areas (LGAs) in nine states in Nigeria, compared with 101 cases reported from 70 LGAs in 13 states during the same period in 2012. For all of 2012, a total of 122 cases were reported. No WPV type 3 (WPV3) cases have been reported since November 2012. For the first time ever, in 2013, no polio cases of any type have been detected in the northwest of Nigeria; however, transmission continues in Kano and states in the northeast. Despite considerable progress, 24 LGAs in 2012 and seven LGAs in 2013 reported two or more cases; WPV continues to circulate in eight LGAs that had cases in 2012. Efforts to interrupt transmission remain impeded by insecurity, anti–polio-vaccine sentiment, and chronically poor SIA implementation in selected areas. Improvement of SIA quality and effective outbreak response will be needed to interrupt WPV transmission in 2014.

## Vaccination Activities

Increasing routine immunization coverage is a key polio eradication strategy. Reported administrative vaccine data indicate that national coverage with 3 doses of trivalent oral polio vaccine (OPV3) increased from 73% in 2012 to 84% during January–September 2013. Among children aged 6–35 months with nonpolio acute flaccid paralysis (AFP), the proportion with a dose history of ≥4 OPV doses, nationwide, increased from 75% in 2012 to 87% in 2013.

Fifteen SIAs* were implemented during January 2012–September 2013; four national rounds of SIAs used trivalent (type 1, type 2, and type 3) oral polio vaccine (OPV), and 11 subnational rounds used bivalent (type 1 and type 3) OPV in high-risk northern states. Vaccination rounds in January and June 2013 were limited in scope, focusing on persistently poor-performing areas and on states with recent polio cases. In February 2013, Nigeria’s polio eradication program suffered setbacks when 13 health workers were targeted and killed in separate attacks in Borno and Kano, resulting in suspension of an SIA and cancelation of the follow-up round. Terrorist attacks had further negative impact on planned SIAs in Yobe and Borno, and some SIAs were repeatedly missed in both states. Borno participated in seven of the eight scheduled SIAs in 2013. When Borno was included, a substantial proportion (37%–55%) of the 27 LGAs did not take part, and some SIAs were of poor quality, as indicated by postcampaign lot quality assurance sampling (LQAS) surveys. To extend reach, OPV vaccination was added to several subnational campaigns with other vaccinations in 2013, including campaigns with measles and serogroup A meningococcal conjugate vaccines (4).

**FIGURE 1:**
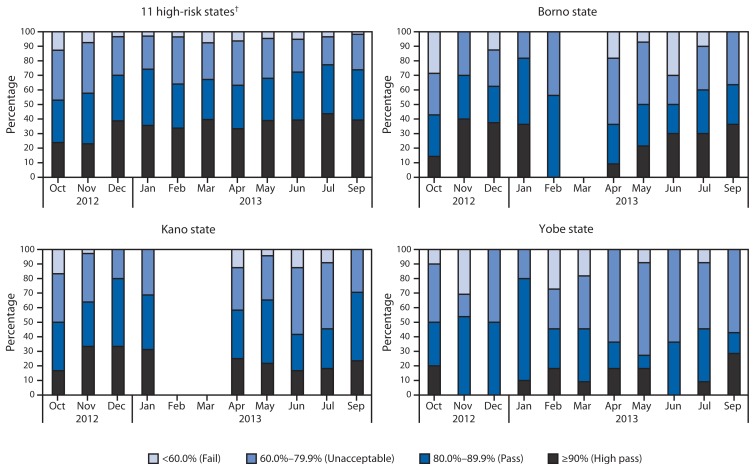
Percentage of local government areas with indicated quality category from lot quality assurance sampling (LQAS^*^) surveys assessing supplementary immunization activities, by month— northern Nigeria, October 2012–September 2013 ^*^ LQAS surveys are used to assess the quality of polio supplemental immunization activities (SIAs) in local government areas, using a four-category pass/fail scheme based on the proportion of children with a finger mark indicating they had received oral polio vaccine during the SIA. ^†^ Bauchi, Borno, Jigawa, Kaduna, Kano, Katsina, Kebbi, Niger, Sokoto, Yobe, and Zamfara.

SIA quality in LGAs is assessed through LQAS surveys using a four-category pass/fail scheme based on the proportion of children with a finger mark (indicating they received OPV during the SIA) (2).† During February 2012–September 2013, the number of LGAs conducting LQAS among the 11 high-risk states§ increased from 87 to 168. During February 2012–September 2013, the proportion of LGAs conducting LQAS in the 11 high-risk states with SIA assessments meeting the ≥90% range increased from 7% to 39%, and the proportion at the 80%–89% range increased from 9% to 35%. The proportion of LGAs at the 60%–79% range decreased from 43% to 24%, and the proportion at the <60% range declined from 40% to 2%. As noted, some LGAs in Borno and Yobe did not conduct any SIAs during this period because of insecurity. In addition, during October 2012–February 2013, when SIAs were held, less overall improvement was noted in these states suggesting insecurity negatively impacted quality. This also was noted in Kano, where LQAs results decreased for several SIAs after February 2013, when polio workers were attacked (Figure 1).

## Poliovirus Surveillance

A nonpolio AFP rate of ≥2 cases per 100,000 children aged <15 years and collection of adequate stool specimens in ≥80% of AFP cases are key performance indicators for AFP surveillance. In 2013, the nonpolio AFP rate was 8.8 cases per 100,000, and 86.5% of AFP cases had adequate stool specimens collected, although these indicators decreased slightly from 2012. The proportion of high-risk states that met both indicators increased from 82% in 2012 to 91% in 2013. Within these states, the proportion of LGAs meeting both requirements increased from 67% in 2012 to 75% in 2013.

AFP surveillance is supplemented by environmental surveillance, for which sewage samples taken every 4–5 weeks are tested for polioviruses. Environmental surveillance in Nigeria was expanded from three sites in 2011 in Kano to 19 sites in 2013: Borno (three sites), Kaduna (two), Kano (three), Lagos (five), Sokoto (four), and the Federal Capital Territory (FCT) (two). During January–September 2013, WPV type 1 (WPV1) was identified in three positive sewage samples (one from Kano in a sample collected in February and two from Sokoto samples collected in March and April). In 2012, WPV1 was identified in two positive sewage samples from Kano (September and October), whereas Sokoto had 16 WPV1 positive sewage samples through September 30, 2012, and none during October–December 2012.

## Wild Poliovirus Incidence

**FIGURE 2:**
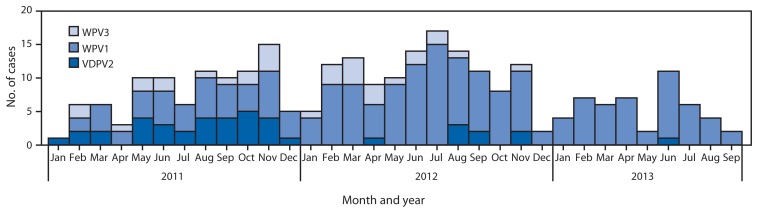
Number of cases of wild poliovirus type 1 (WPV1), wild poliovirus type 3 (WPV3), and vaccine-derived poliovirus type 2 (VDPV2), by month — Nigeria, January 2011–September 2013

**FIGURE 3:**
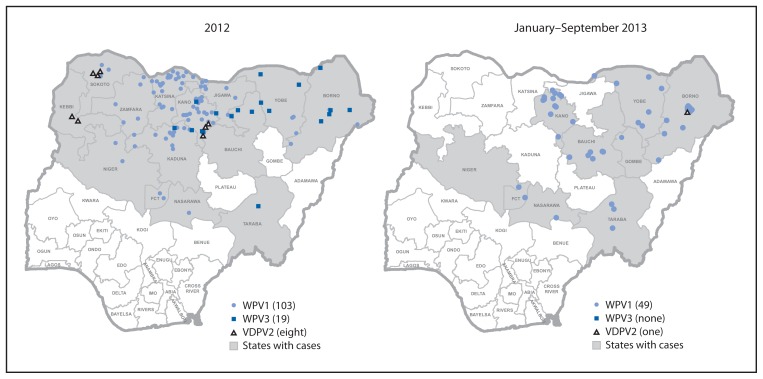
Distribution of cases of wild poliovirus type 1 (WPV1), wild poliovirus type 3 (WPV3), and vaccine-derived poliovirus type 2 (VDPV2), by month — Nigeria, 2012 and January–September 2013^*^ ^*^ Each dot represents one WPV case placed at random within a local government area boundary.

The number of WPV cases in Nigeria increased from 62 in 2011 to 122 in 2012. From January to September in 2012, a total of 101 cases were reported, compared with 49 cases reported during the same period in 2013 (as of November 27, 2013). These case counts remain higher than 2010 levels, when 21 cases were reported through September (2). No WPV3 cases have been reported in Nigeria since November 2012 (Figure 2). Early in 2013, cases were reported in previously unaffected LGAs in the north central states of Nassarawa, Niger, and FCT. More recently, WPV transmission shifted geographically from the northwest part of the country to the northeast (Figure 3). Forty-two of 49 cases in 2013 (86%) were reported from Borno (16 cases), Kano (13), Yobe (seven), and Bauchi (six). Compared with 2012, the number of affected states declined from 13 to nine, and the number of affected LGAs dropped from 70 to 26. Eight cases of circulating vaccine-derived poliovirus type 2 (cVDPV2) were reported in 2012 and one cVDPV2 case has been reported in 2013 (onset of paralysis on June 6).

As poliovirus circulation declines in reservoir communities, the genetic diversity of poliovirus isolates also declines. The number of genetic clusters¶ circulating in 2013 will be determined in early 2014 based on genetic analysis of all 2013 cases. Two genetic clusters present in 2012 also have been found in 2013. In 2011, 11 WPV1 clusters were circulating; eight continued to circulate in 2012. Partial genomic sequence analysis also is used to assess surveillance sensitivity; a nucleotide difference of ≥1.5% in the coding region of the major capsid protein, VP1, from the closest matching sequences of previously identified isolates indicate gaps in surveillance with >1 year of undetected virus circulation (1,8). Of 100 WPV cases detected during January–September 2012, 13 (13%) had less genetic linkage than expected with sensitive AFP surveillance, compared with eight (16%) of the 49 cases detected in 2013.

Genomic sequence analysis indicated that the cVDPV2 case identified in Borno in 2013 was closely related to a cVDPV2 lineage that had circulated in Chad in 2012 and spread to neighboring countries such as Cameroon and Niger. In addition, sequence analysis indicated that WPV1 isolates from environmental samples in Sokoto in March and April 2013 were not of the same cluster circulating in the area in 2012, but rather were related to a cluster circulating in other states.

### Editorial Note

Considerable progress has been made in Nigeria toward polio eradication. As of late November 2013, no WPV 3 cases had been reported for more than a year; the geographic extent of WPV transmission appears to no longer include the western-most states, and the number of WPV cases reported through September 2013 (49) was half that reported during the same period in 2012 (101). Despite these successes, continued WPV1 transmission in Nigeria is a threat to global polio eradication and must be addressed urgently, particularly as the country enters the relatively low WPV transmission season (November–April).

Several strategies are being employed to improve program performance and address specific constraints in LGAs defined as high-risk by SIA evaluation data (LQAS) and statistical modeling. To address anti–polio vaccination sentiment and the threat of violence, social and community mobilization activities provide opportunities for community leaders to engage and become advocates for the protection of children against the acquisition of poliovirus. To enhance community engagement where noncompliance has been particularly high, >1,000 polio survivors work to raise risk perception, and health camps (temporary mobile health service stations) are held during SIAs to address unmet primary health-care needs. Approximately 200 community outreach workers have engaged with religious leaders and Koranic school teachers in high-risk LGAs to further enhance community support. Additionally, several strategies are used to enhance campaign performance including: 1) interagency “management support teams” deployed at the ward level to assist in the supervision of SIA activities; 2) “management and accountability officers” who monitor funding expenditures and increase local accountability; and 3) global postitioning system (GPS) tracking that is used to improve microplanning and track vaccination teams during SIAs.

Nigeria experienced setbacks in 2013, including continued low SIA quality in specific areas and states, the targeted killing of polio workers, and high levels of insecurity in the northeast. Despite increased political commitment and accountability in Kano, persistently poor performing LGAs remain throughout much of the state. Activities taken to address the substantial immunity gap among children living in security-compromised areas of Borno where SIAs could not be conducted include 1) mobile vaccination teams, 2) intensified routine infant immunization and other health services, 3) shortened intervals between OPV doses, 4) health camps coupled with intensified community engagement, and 5) vaccination posts placed at state and LGA borders to vaccinate children in transit. In addition, operational funds and prepositioned vaccine stocks are being provided so that SIAs can be organized quickly and implemented when windows of opportunity open for safe deployment of vaccination teams.

What is already known on this topic?

Nigeria is the only country in Africa where wild poliovirus (WPV) transmission has never been interrupted and has served as a reservoir for polio outbreaks throughout the last decade. Low routine immunization coverage, poor quality supplemental immunization activities (SIA), and anti–polio-vaccination sentiment have historically provided opportunities for continued virus transmission throughout the northern part of the country. To address ongoing WPV transmission, the Nigerian government restructured the national polio response in 2012 and increased efforts to reach missed children through routine immunization and improved SIA.

What is added by this report?

During January–September 2013, 49 polio cases were reported from 26 local government areas (LGAs) in nine states, compared with 101 cases reported from 70 LGAs in 13 states during the same period in 2012. No cases have been detected in the most northwestern states for the first time ever, but transmission continues in Kano and states in the northeast where high levels of insecurity, violence targeting polio workers early in 2013, and continued suboptimal SIA planning continue to impede progress. In addition, there are indications of substantial surveillance gaps that need to be addressed.

What are the implications for public health practice?

Global polio eradication efforts have long been challenged by WPV circulation in Nigeria. Unless further improvements are made in improved SIA quality and effective outbreak response, there is considerable risk of not interrupting WPV transmission in 2014. A comprehensive immunization strategy and improvements in campaign implementation will be needed to reach all unimmunized children while increasing community demand for routine vaccination.

Despite the setbacks, Nigeria has made considerable progress toward polio eradication in the last 18 months. Successful interruption of WPV transmission will depend on a sustained focus on the issues raised and effective outbreak response. Virologic analysis revealed that substantial gaps remain in AFP surveillance, which must be strengthened to further pinpoint poliovirus circulation. Enhanced AFP surveillance, along with environmental surveillance, can subsequently document that transmission is interrupted when cases are no longer detected.

## Footnotes

* Mass campaigns conducted for a brief period (days to weeks) in which 1 dose of OPV is administered to all children aged <5 years, regardless of vaccination history. Campaigns can be conducted nationally or in portions of the country.

† A clustered LQAS methodology is used to assess SIA quality by sampling the target population of children at the LGA level and documenting finger markings indicative of OPV receipt. A sample is drawn from six wards (geopolitical subunits) within the LGA, with 10 children in a single settlement selected at random from each sampled ward, yielding a sample of 60 children per LGA. SIAs in the LGAs are classified into one of four quality categories based on the number of unmarked children found: 0–3, tested range >90.0% (high pass); 4–8, tested range 80.0%–89.9%, (pass); 9–19, tested range ≥60.0%–79.9% (unacceptable); and >19, tested range <60.0% (fail). A detailed description of the methodology is available at http://www.polioeradication.org/portals/0/document/research/opvdelivery/lqas.pdf.

§ Bauchi, Borno, Jigawa, Kaduna, Kano, Katsina, Kebbi, Niger, Sokoto, Yobe, and Zamfara.

¶ VP1, the ~900 nucleotide major capsid protein of WPV, is sequenced on all WPV isolates globally to assess viral transmission. Genetic clusters consist of WPV isolates with >95% VP1 nucleotide identity.
